# Estrogen markedly reduces circulating low-density neutrophils and enhances pro-tumoral gene expression in neutrophil of tumour-bearing mice

**DOI:** 10.1186/s12885-021-08751-2

**Published:** 2021-09-11

**Authors:** Chew Leng Lim, Valerie C.-L. Lin

**Affiliations:** 1 NTU Institute for Health Technologies, Interdisciplinary Graduate School, Nanyang Technological University, Singapore, Singapore; 2 School of Biological Sciences, Nanyang Technological University, Singapore, Singapore

**Keywords:** Neutrophils, Mammary involution, Estrogen

## Abstract

**Background:**

Neutrophils are important for immune surveillance of tumour cells. Neutrophils may also be epigenetically programmed in the tumour microenvironment to promote tumour progression. In addition to the commonly known high-density neutrophils (HDN) based on their separation on density gradient, recent studies have reported the presence of high levels of low-density neutrophils (LDN) in tumour-bearing mice and cancer patients. We reported previously that estrogen promotes the growth of estrogen receptor α-negative mammary tumours in mice undergoing mammary involution through stimulating pro-tumoral activities of neutrophils in the mammary tissue.

**Methods:**

Female BALB/cAnNTac mice at 7–8 weeks old were mated and bilateral ovariectomy was performed 2 days post-partum. At 24 h after forced-weaning of pups to induce mammary involution, post-partum female mice were injected with either E2V, or vehicle control on alternative days for 2-weeks. On 48 h post-weaning, treated female mice were inoculated subcutaneously with 4 T1-Luc2 cells into the 9th abdominal mammary gland. Age-matched nulliparous female was treated similarly. Animals were euthanized on day 14 post-tumour inoculation for analysis. To evaluate the short-term effect of estrogen, post-partum females were treated with only one dose of E2V on day 12 post-tumour inoculation.

**Results:**

Estrogen treatment for 2-weeks reduces the number of blood LDN by more than 10-fold in tumour-bearing nulliparous and involuting mice, whilst it had no significant effect on blood HDN. The effect on tumour-bearing mice is associated with reduced number of mitotic neutrophils in the bone marrow and increased apoptosis in blood neutrophils. Since estrogen enhanced tumour growth in involuting mice, but not in nulliparous mice, we assessed the effect of estrogen on the gene expression associated with pro-tumoral activities of neutrophils. Whilst 48 h treatment with estrogen had no effect, 2-weeks treatment significantly increased the expression of *Arg1*, *Il1b* and *Tgfb1* in both HDN and LDN of involuting mice. In contrast, estrogen increased the expression of *Arg1* and *Ccl5* in HDN and LDN of nulliparous mice.

**Conclusions:**

Prolonged estrogenic stimulation in tumour-bearing mice markedly hampered tumour-associated increase of LDN plausibly by inhibiting their output from the bone marrow and by shortening their life span. Estrogen also alters the gene expression in neutrophils that is not seen in tumour-free mice. The results imply that estrogen may significantly influence the tumour-modulating activity of blood neutrophils.

**Supplementary Information:**

The online version contains supplementary material available at 10.1186/s12885-021-08751-2.

## Introduction

Neutrophils belong to the innate immune system that is important for modulating immune response, defence against pathogens, and wound healing [1]. Over the years, the involvement of neutrophils in tumour development has gained recognition. Following the classification of tumour-associated M1 and M2 macrophages, neutrophils were also classified into the anti-tumour (N1) and pro-tumour neutrophils (N2) based on cellular morphology and gene expression profile [2, 3]. Another subset of immune cells known as the myeloid derived suppressor cells (MDSC) expands in response to conditions such as chronic infection, autoimmune diseases, and cancer [4]. MDSCs consists of granulocytic MDSC (g-MDSC) which are morphologically and phenotypically similar to neutrophils, and monocytic MDSC (m-MDSC) which display phenotypes similar to monocytes. It is widely accepted that the immunosuppressive activities of MDSCs favour tumour progression [5]. Neutrophils can also be classified into the commonly known high-density neutrophils (HDN) and low-density neutrophils (LDN) based on their density separation in a discontinuous density gradient. The LDN was originally reported in patients with auto-immune conditions [6]. It is now known to accumulate in tumour-bearing mice and cancer patients with immunosuppressive activities [7–9]. LDN was reported to be comprised of neutrophils displaying both immature phenotype akin to the g-MDSC and the mature neutrophils, whereas HDN are neutrophils mostly of mature phenotype and are cytotoxic to tumour cells [7].

It is well-established that estrogen is the primary driver of estrogen receptor α (ERα)-positive breast cancer which accounts for up to 70% of all breast cancer cases [10]. Estrogen deprivation therapies such as anti-estrogen and aromatase inhibitors are the front-line therapy for ERα-positive breast cancers. It is also understood in recent years that estrogen can promote the progression of ER-negative breast cancer by influencing the tumour microenvironment. Estrogen promotes tumour development through mobilization of MDSCs and their accumulation in tumour tissue [11, 12]. In mouse xenograft models of immunocompetent mice and nude mice, estrogen stimulated the infiltration of neutrophils to the invasive edge of mammary tumours, and estrogen-stimulated expression of lymphocyte function-associated antigen 1 (LFA-1) plays a critical role in tumour cell metastasis [13]. These emerging evidences indicates that estrogen regulates neutrophils activity to create a pro-tumoral microenvironment. There is evidence that pro-tumoral effect of estrogen on neutrophils is mediated by ERα [12]. On the other hand, ERβ-specific ligand LY500307 has been reported to induce neutrophils infiltration to the tumour and suppress cancer metastasis [14]. Thus, estrogen can influence tumour biology through modulating the activities of neutrophils but the outcome depends on the pathological context.

We have previously reported that in BALB/cAnNTac mice undergoing post-weaning mammary involution (Inv), estrogen promoted neutrophil infiltration into the mammary tissue. The estrogen-induced neutrophil infiltration plays a significant role in the tumour growth as the depletion of neutrophils reduced estrogen-induced tumour growth [15]. This effect appears to be mediated by estrogen-induced expression of cytokines/chemokines in mammary neutrophils that contribute to the pro-tumoral microenvironment. However, whether estrogen influences the activity of circulating neutrophils is not known.

The present study investigates the effect of estrogen on the levels and activities of circulating HDN and LDN in relation to tumour development. Our data show that prolonged estrogen treatment markedly reduces the number of circulating LDN in tumour-bearing Inv and nulliparous (Null) mice. This is associated with reduction in mitotic neutrophils in the bone marrow and increased apoptosis of blood neutrophils. The results suggest that estrogen exerts significant influence on the levels of circulating LDN and upregulates the expression of genes associated with pro-tumoral neutrophils.

## Materials and methods

### Animal studies

All animal experiments were performed in accordance with the protocol approved by the Nanyang Technological University Institutional Animal Care and Use Committee (NTU-IACUC) (IACUC protocol number: A0306 and A18036). The study was performed in compliance with the ARRIVE ESSENTIAL 10 guidelines. BALB/cAnNTac mice used in the study were housed in specific pathogen-free facility under a 12 h dark/light cycle and provided food and water ad libitum. Female mice at 7 to 8 weeks old were mated and housed individually. Bilateral ovariectomy (OVX) was performed 2 days post-partum. Litter sizes were standardized to 5–6 pups per lactating female. Null females were handled similarly as the pregnant mice. All mice were randomly distributed into the treatment groups.

### Tumour study

Fourteen days post-partum (L14), pups were weaned to induce mammary involution. At 24 h post-weaning (Inv D1), post-partum females were injected subcutaneously with either estradiol valerate (E2V) (Selleck Chemicals), at 100 μg/kg body weight, or sesame oil as vehicle control (Ctrl) once every 2 days for 2-weeks. On 48 h post-weaning (Inv D2), treated mice were anaesthetized and inoculated subcutaneously with 1 million 4 T1-Luc2 cells into the 9th abdominal mammary gland using a 26-gauge needle. Age-matched OVX Null female was treated similarly. To evaluate the short-term effect of estrogen, post-partum females were instead treated with only one dose of E2V on day 12 post-tumour inoculation. All animals were euthanized on day 14 post-tumour inoculation for analysis.

### Isolation of blood LDN and HDN

Blood collected via cardiac puncture was layered over a discontinuous Percoll (Sigma-Aldrich) gradient and centrifuged at 1000 g for 35 min, 22 °C, with no acceleration and brake. Cells at the 55 and 65% interface were collected as mononuclear cell layer while cells at the 65 and 80% interface were collected as granulocyte layer. Red blood cells (RBCs) were lysed with 5 ml 0.2% (w/v) NaCl, and the lysis stopped by adding 5 ml 1.6% (w/v) NaCl. Cells were pelleted and resuspended in 1xPBS. The number of HDN and LDN per ml of blood were determined by deriving the number of cells/ml in the Percoll isolated mononuclear layer and granulocyte layer, followed by multiplying with the percentage of CD45^+^CD11b^+^Ly6G^+^ cells obtained from flow cytometry analysis of the same isolated layer. As we assume that LDN and HDN make up the entire population of blood neutrophils, the number of total circulating neutrophils per ml of blood is the sum of LDN and HDN number.

Cells in the granulocyte layer of the gradient were collected directly for flow cytometry and RNA analysis. Cells in the mononuclear layer were stained with PE-anti-Ly6G (clone 1A8), APC-Cy7-anti-CD45 (clone 30-F11), and FITC-anti-Ly6C (clone HK1.4) at 1:200 dilution. LDN (CD45^+^Ly6G^+^Ly6C^int^) was sorted by fluorescence-activated cell sorting (FACS). Sorted LDN was pelleted and lysed with TRIzol for RNA extraction.

### Isolation of tumour associated immune cells

A portion of the inoculated tumour collected from the euthanized mice were minced into small pieces of approximately 1 mm. Minced tumour were then digested in phenol-red free Dulbecco’s Modified Eagle Medium (DMEM) (Nacalai Tesque) containing 2 mM L-glutamine (GE healthcare), 1 mg/ml collagenase (Sigma-Aldrich), and 120 Kunitz DNase I (Sigma-Aldrich) for 1 h at 37 °C in water bath with agitation. Digestion was inactivated with an equal volume of medium and cells were sieved through a 100 μm sieve and centrifuged at 450 g for 5 min at 4 °C. Red blood cells (RBC) lysis was then performed with 1 ml NH_4_Cl buffer (1 volume of 0.17 M Tris-HCL and 9 volume of 0.155 M NH_4_Cl). After inactivation of RBC lysis with 10 volume of 1xPBS, cells were pellet and used for flow cytometry analysis.

### Isolation of bone marrow cells

Bone marrow cells were flushed out from the medullary cavity of the femurs and tibias with 1xPBS using syringe and needle. Isolated bone marrow cells were pelleted at 500 g for 10 min at 4 °C and removed of supernatant. RBCs was lysed by incubation with NH_4_Cl buffer (1 volume 0.17 M Tris-HCL and 9 volume 0.155 M NH_4_Cl) followed by inactivation with 1xPBS. Cells were then pelleted and resuspended in 1xPBS for further analysis.

### Isolation of splenic immune cells

The spleen was smashed using syringe plunger and sieved through a 70 μm mesh. The flow through splenic ells were pelleted at 500 g for 10 min at 4 °C and resuspended with NH_4_Cl buffer to lyse RBCs. After inactivation of RBC lysis with 1xPBS, cells were pelleted and resuspended in 1xPBS.

### Flow cytometry analysis

Staining of cells was carried out with a cocktail of primary antibodies on ice at 1:200 dilution from either BioLegend or eBioscience comprising of APC-Cy7-anti-CD45 (clone 30-F11), FITC-anti-Ly6C (clone HK1.4), PE-anti-Ly6G (clone 1A8), BV605-anti-CD11b (clone M1/70), FITC-anti-TCRγ/δ (clone GL3), PerCP/Cy5.5-anti-CD4 (clone GK1.5), and BV605-anti-CD8a (clone 53–6.7). Dead cells were stained with eFluor 450-fixable viability dye (eBioscience) at 1:1000 dilution before fixation with fixative buffer (BioLegend). The gating strategy of flow cytometry analysis is outline in Supplementary Figure 1A.

### Annexin V staining for flow cytometry analysis

Cells were stained for surface markers and dead cells before washing with 1x annexin buffer (Thermo Fisher Scientific). Samples were then incubated with Alexa Fluor® 488-AnnexinV antibody (Thermo Fisher Scientific) diluted at 1:20 with 1x annexin buffer at room temperature. Cells were then washed with 1x annexin buffer and fixed at room temperature before resuspended in 1x annexin buffer for analysis. The gating strategy of flow cytometry analysis is outline in Supplementary Figure 1B.

### BrdU staining for flow cytometry analysis

5-bromo-2′-deoxyuridine (BrdU) dissolved in 1xPBS was administered at 0.5 mg per mice via intraperitoneal injection 24 h prior to euthanasia. One million cells were used for BrdU staining. Surface antigen were stained using a cocktail of PE-anti-Ly6G (clone 1A8), APC-Cy7-anti-CD45 (clone 30-F11), and BV605-anti-CD11b (clone M1/70) (1:200) on ice. Cells were washed with PBS and fixed with 4% paraformaldehyde before were permeabilized with 0.5% (v/v) Triton-X100 (Bio-Rad) in 1xPBS. Cells were treated with 20 μg of DNase I in 1xHBSS (with calcium and magnesium) at 37 °C before stained with Alexa Fluor® 488-anti-BrdU (BioLegend) (1:100) at room temperature. Finally, cells were pelleted and resuspended in propidium iodide (PI) solution at 0.5 μg/ml for analysis.

### Quantitative polymerase chain reaction (qPCR)

Total RNA was extracted from cells using TRIzol reagent (Life technologies). Extracted RNA was reverse transcribed into cDNA using qScript cDNA SuperMix (Quantabio). qPCR was carried out with KAPA SYBR FAST qPCR Master Mix (KAPA Biosystems) on the Quantstudio 6 Flex Real-Time PCR System (Applied Biosystems). qPCR for each target gene was performed in duplicates. For quantitative analysis, the comparative Threshold Cycle (*C*_t_) were calculated against the *C*_t_ value of *Gapdh* as the internal control. Relative quantification was performed using the 2^−Δ*C*t^ method [16] and expressed as relative mRNA level in arbitrary values. Primers are *Arg1*: F-ggaatctgcatgggcaacctgtgt, R-agggtctacgtctcgcaagcca; *Ccl5*: F- accatgaagatctctgcagc, R-tgaacccacttcttctctgg; *Gapdh*: F-tgcaccaccaactgcttag, R-gaggcagggatgatgttc; *Il1b*: F-caaccaacaagtgatattctccatg, R-gatccacactctccagctgca; *Tgfb1*: F-gaccgcaacaacgccatcta, R-gttccacatgttgctccacact.

### Statistical analysis

Graphs were plotted using the mean value with the standard error of the mean (SEM). When comparing 2 groups, statistical significance was determined using a two-tailed unpaired student’s T-test. When comparing between more than 2 groups, one-way ANOVA followed by post-hoc Tukey test was performed. All statistical analysis was performed using the GraphPad Prism 7 software; *p*-value: < 0.05 (*), < 0.01 (**), < 0.001 (***), < 0.0001 (****). Statistical analysis was considered significant with the *p*-value < 0.05.

## Results

### Estrogen promotes the growth of mammary tumour in mice undergoing mammary involution but not in nulliparous mice

**Fig. 1:**
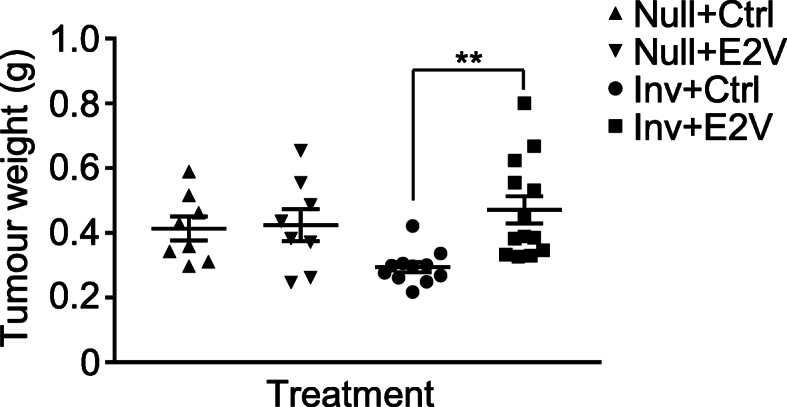
Estrogen induced increased growth of ER-negative tumour during mammary involution. From 24 h post-weaning (Inv D1), OVX Inv mice were treated with estradiol valerate (E2V) or vehicle control (Ctrl). Mice were subsequently inoculated with 4 T1-Luc2 cells at the 9th abdominal mammary gland on Inv D2. Age-matched nulliparous (Null) were treated similarly. Mice were euthanized and tumour harvested at day 14 post-tumour cells inoculation. Estrogen treatment induces increased tumour growth in involuting mice but not in nulliparous; Tumour weight at 14 days post-inoculation, Null+Ctrl *n* = 8, Null+E2V *n* = 8, Inv + Ctrl *n* = 11, Inv + E2V *n* = 13. Data represented as mean ± SEM. An outlier in Inv + Ctrl group was identified using Grubbs’ test and was removed. Statistical significances determined using two-tailed unpaired student’s T-test

We have reported previously that estrogen promotes ERα-negative mammary tumour growth in BALB/cAnNTac mice undergoing mammary involution [15]. In this study, we compared the effect of estrogen on tumour growth in Inv mice and age-matched Null. In contrast to the Inv mice, where estradiol valerate (E2V) stimulated tumour growth significantly, E2V treatment in Null mice had no significant effect on the weight of 4 T1-Luc2 tumour (Fig. 1). This suggests that tissue microenvironment during mammary involution is necessary for estrogen to stimulate the growth of ERα-negative mammary tumour. Estrogen-stimulated neutrophils contribute to the tissue microenvironment in the mammary tissue [15]. In the following studies, we determined how estrogen influences the activities of circulating neutrophils.

### Estrogen significantly reduces circulating neutrophils in tumour-bearing mice

**Fig. 2:**
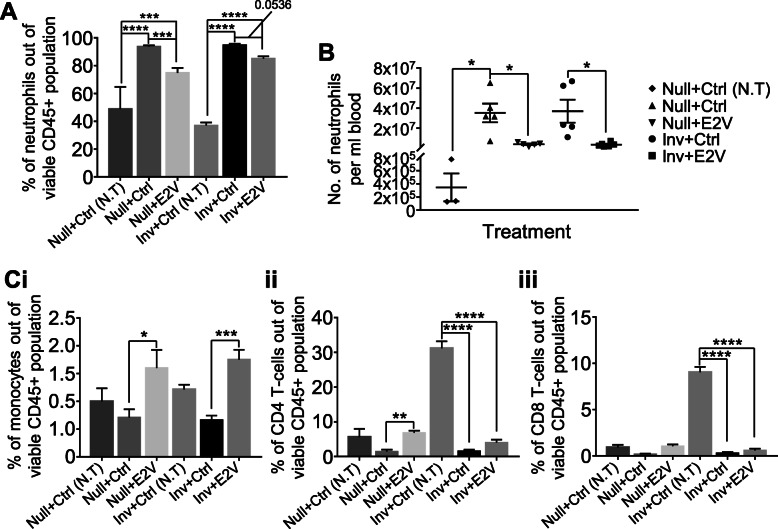
Estrogen reduces circulating neutrophil in tumour-bearing mice. Experiment is conducted according to the description in Fig. 1. **A**, Tumour burden increases circulating neutrophils which were reduced with estrogen treatment in both Inv and Null mice; Percentage of circulating neutrophils (CD45^+^CD11b^+^Ly6G^+^) out of viable CD45^+^ population via flow cytometry analysis, Null+Ctrl (no tumour - N.T) *n* = 3, Null+Ctrl *n* = 8, Null+E2V *n* = 8, Inv + Ctrl (N.T) *n* = 6, Inv + Ctrl *n* = 12, Inv + E2V *n* = 13. **B**, Number of neutrophils per ml of blood, Null+Ctrl (N.T) *n* = 3, Null+Ctrl *n* = 5, Null+E2V *n* = 5, Inv + Ctrl *n* = 5, Inv + E2V *n* = 6. **C**, Estrogen-induced reduction of circulating neutrophils in tumour-bearing mice were not due to increase in other circulating leukocytes; (i) Percentage of circulating monocytes (CD45^+^CD11b^+^Ly6C^hi^) out of viable CD45^+^ population, (ii) Percentage of circulating CD4 T-cells (CD45^+^CD4^+^CD8^−^) out of viable CD45^+^ population, (iii) Percentage of circulating CD8 T-cells (CD45^+^CD8^+^CD4^−^) out of viable CD45^+^ population via flow cytometry analysis, Null+Ctrl (N.T) *n* = 3, Null+Ctrl *n* = 8, Null+E2V *n* = 8, Inv + Ctrl (N.T) *n* = 6, Inv + Ctrl *n* = 12, Inv + E2V *n* = 13. Data represented as mean ± SEM. Statistical significances determined using one-way ANOVA followed by post-hoc tukey test

OVX mice inoculated with tumour were treated with control vehicle (Ctrl) or E2V on alternative days. Blood samples were analysed on day 14 post-tumour cell inoculation. The levels of circulating neutrophils were evaluated by flow cytometry. The percentage of circulating neutrophils (CD45^+^CD11b^+^Ly6G^+^) doubled in mice with tumour compared to tumour-free mice under both Null and Inv conditions (Fig. 2A, Supplementary Figure 2). More interestingly, treatment with E2V resulted in marked reductions of the circulating neutrophils in both tumour-bearing Null and Inv mice. To confirm that the reduction of neutrophils percentage in response to E2V is due to decrease of absolute number of neutrophils, the number of neutrophils per ml blood was determined by cell counting. E2V reduced the number of neutrophils per ml by ~ 10 times (Fig. 2B; Null+Ctrl, 35.2 × 10^7^/ml; Null+E2V, 3.8 × 10^7^/ml; Inv + Ctrl, 36.9 × 10^7^/ml; Inv + E2V, 3.5 × 10^7^/ml). There are also significant increases in the percentage of circulating monocytes (CD45^+^CD11b^+^Ly6C^hi^), CD4 T-cells (CD45^+^CD4^+^CD8^−^), and CD8 T-cells (CD45^+^CD8^+^CD4^−^) out of CD45^+^ cells in the tumour-bearing mice with E2V. We believe that these are likely due to decreases of the proportion of neutrophils (Fig. 2C, Supplementary Figure 3). Interestingly, we also observed that the proportion of CD4 and CD8 T-cells in non-tumour Inv mice is 5 times higher than that in non-tumour Null mice. Why such a difference exists is not known.

### Estrogen-induced reduction of circulating neutrophils in tumour-bearing mice is due to reduction in LDN

**Fig. 3:**
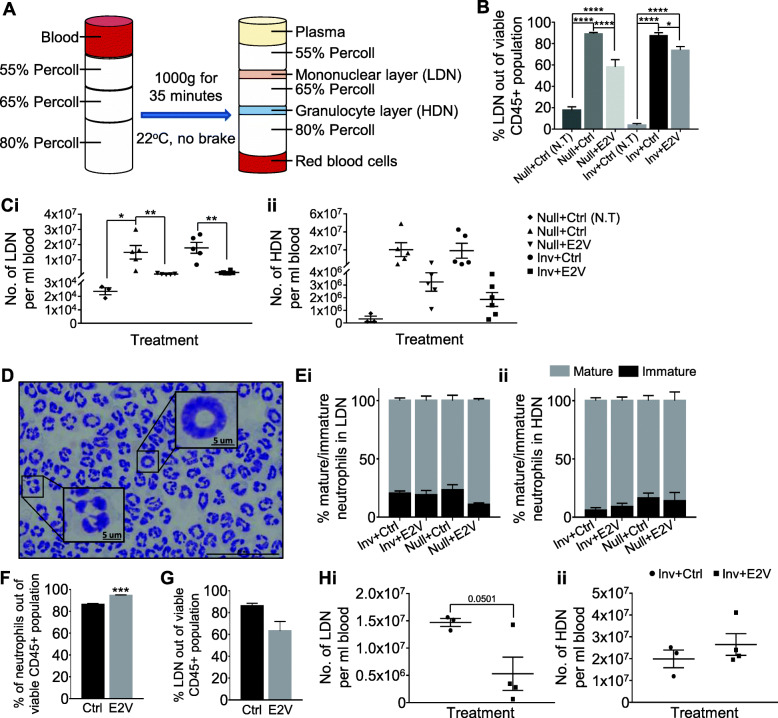
Estrogen reduces circulating low-density neutrophils (LDN) in tumour-bearing mice. **A**, Schematic diagram of the discontinuous Percoll gradient used for the separation of blood into the different cell fractions. **B-E**, Experiment is conducted according to the description in Fig. 1. **B**, Percentage of circulating LDN (CD45^+^CD11b^+^Ly6G^+^) out of viable CD45^+^ population via flow cytometry analysis, Null+Ctrl (N.T) *n* = 3, Null+Ctrl *n* = 8, Null+E2V *n* = 8, Inv + Ctrl (N.T) *n* = 6, Inv + Ctrl *n* = 12, Inv + E2V *n* = 13. **C**, Estrogen treatment significantly reduces the amount of circulating LDN in tumour-bearing mice while not significantly affecting circulating HDN; (i) Calculated number of circulating LDN per ml blood, (ii) Calculated number of circulating HDN per ml blood. Null+Ctrl (N.T) *n* = 3, Null+Ctrl *n* = 5, Null+E2V *n* = 5, Inv + Ctrl *n* = 5, Inv + E2V *n* = 6. D, Representative image of Giemsa stained mature (multilobed nucleus) and immature (banded nucleus) neutrophils. Scale bar: 50um. **E**, Estrogen treatment in tumour-bearing mice does not affect the proportion of mature and immature neutrophils in circulating LDN; (i) Distribution of circulating LDN displaying banded or multilobed nucleus, (ii) Distribution of circulating HDN displaying banded or multilobed nucleus. Inv + Ctrl *n* = 7, Inv + E2V *n* = 7, Null+Ctrl *n* = 3, Null+E2V *n* = 3. **F-H**, At Inv D2, OVX mice were inoculated with 4 T1-Luc2 cells at the 9th abdominal mammary gland. At 48 h prior to sample collection, mice were treated with either E2V or Ctrl. Mice were euthanized and tumour harvested at day 14 post-tumour inoculation. F, Estrogen treatment for 48 h in tumour-bearing Inv mice led to an increased proportion of total circulating neutrophils; Data are expressed as percentage of circulating neutrophils (CD45^+^CD11b^+^Ly6G^+^) out of viable CD45^+^ population via flow cytometry analysis, Inv + Ctrl *n* = 3, Inv + E2V *n* = 4. **G**, Estrogen treatment for 48 h in tumour-bearing Inv mice resulted in a small reduction in circulating LDN while HDN remain unchanged; Data are expressed as percentage of circulating LDN (CD45^+^CD11b^+^Ly6G^+^) out of viable CD45^+^ population via flow cytometry analysis, Inv + Ctrl *n* = 3, Inv + E2V *n* = 4. **H**, (i) Number of circulating LDN per ml blood, (ii) Number of circulating HDN per ml blood. Data represented as mean ± SEM. Statistical significances determined using one-way ANOVA followed by post-hoc tukey test in (**B**) and (**C**); two-tailed unpaired student’s T-test in (**F**-**H**)

We addressed whether estrogen reduced the levels of HDN, LDN, or both in the tumour bearing mice. Using Percoll gradient centrifugation, blood cells were separated into the mononuclear cells, granulocytes, and RBCs (Fig. 3A). The LDN reported in various disease conditions are present in the mononuclear cell fraction. Flow cytometry analysis of the Percoll isolated mononuclear cell layer revealed an expected increase of circulating LDN (CD45^+^CD11b^+^Ly6G^+^) in tumour-bearing Null and Inv mice as compared to their non-tumour counterpart. Interestingly, this increase in LDN in tumour-bearing mice was significantly reduced by E2V in both percentage and in number per ml (Fig. 3B, C, Supplementary Figure 4). Based on the number of LDN per ml of blood, this reduction was about 10-folds (Inv + Ctrl, 1.8 × 10^7^/ml; Inv + E2V, 1.7 × 10^6^/ml) in Inv and 25-folds (Null+Ctrl, 1.5 × 10^7^/ml; Null+E2V, 5.9 × 10^5^/ml) in Null mice (Fig. 3 Ci). Although there is a decreasing trend, E2V did not significantly decrease the number of HDN in both tumour-bearing Null and Inv mice (Fig. 3Cii; Null, *p* = 0.1858; Inv, *p* = 0.1466). Morphological analysis showed that less than 25% of the isolated LDN are immature neutrophils with banded or ringed nucleus (Fig. 3D, Ei), in contrast to the 65% immature neutrophils reported by Sagiv et al. using the same syngeneic BALB/c-4 T1 tumour model [7]. HDN fraction also had ~ 10% immature neutrophils (Fig. 3Eii). In addition, E2V treatment did not significantly affect the distribution of mature and immature neutrophils in the circulating LDN despite causing marked down-regulation (Fig. 3Ei).

We next evaluated whether the marked down-regulation of LDN is the accumulated effect of the prolonged (14 days) treatment. As similar estrogenic effect was observed in both tumour-bearing Inv and Null mice, we opted to evaluate the effect in the Inv mice only. OVX Inv mice were inoculated with tumour and treated with one dose of E2V at 48 h prior to harvest at 14 days post-tumour inoculation. In contrast to that observed with a 14 days treatment, E2V induced a significant increase in the percentage of circulating neutrophils (Fig. 3F). However, despite the overall increase in proportion, the number of circulating LDN was reduced significantly by 48 h E2V treatment (Fig. 3G, Hi). In contrast, the amount of HDN remain unchanged with E2V treatment (Fig. 3Hii).

Taken together, estrogen treatment for 14 days in tumour-bearing mice resulted in substantial reduction in circulating neutrophils. This reduction is due to the reduction in LDN. Furthermore, this reduction in LDN appears to be accumulative as treatment with E2V for 48 h resulted in a less reduction.

### Estrogen-induced LDN reduction was associated with increased LDN cell death and reduced neutrophil production from bone marrow

**Fig. 4:**
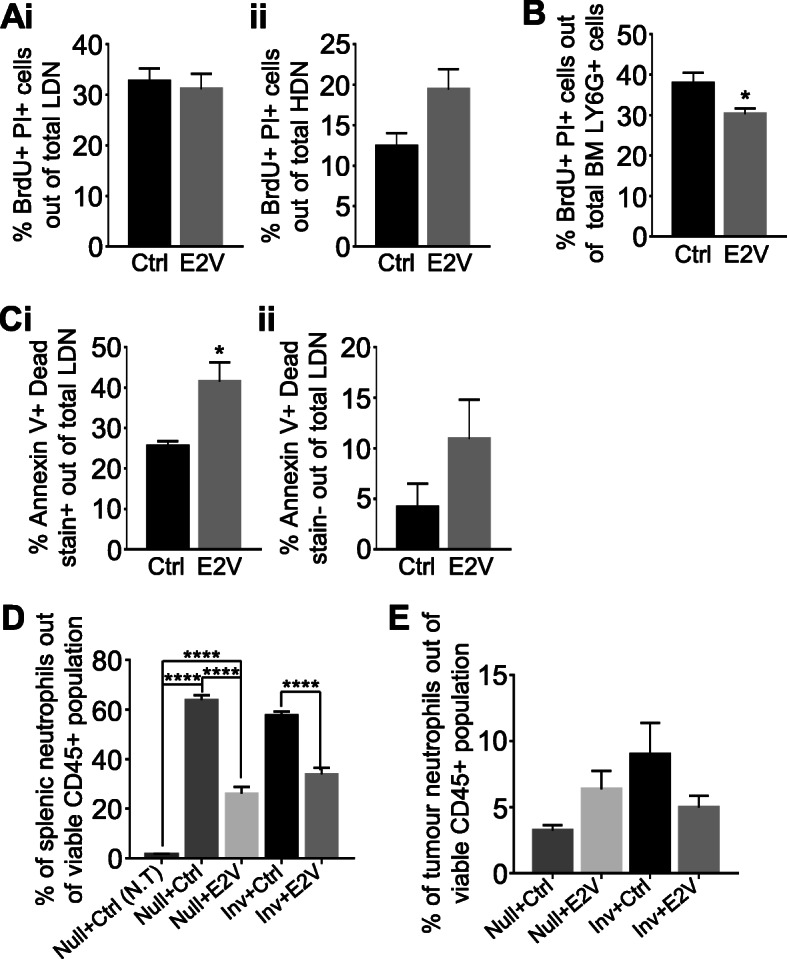
Estrogen treatment increases LDN cell death and reduces bone marrow neutrophil production in tumour-bearing Inv mice. **A**-**C**, At Inv D2, OVX mice were inoculated with 4 T1-Luc2 cells at the 9th abdominal mammary gland. At 48 h prior to sample collection, mice were treated with either E2V or Ctrl. Subsequently, at 24 h before collection, mice were administered with BrdU. Mice were euthanized and tumour harvested at day 14 post-tumour inoculation. **A**, 48 h estrogen treatment did not affect the percentage of newly generated circulating LDN and HDN in the blood; (i) Percentage of BrdU^+^PI^+^ cells out of total circulating LDN (CD45^+^CD11b^+^Ly6G^+^), (ii) Percentage of BrdU^+^PI^+^ cells out of total circulating HDN (CD45^+^CD11b^+^Ly6G^+^) via flow cytometry analysis, Inv + Ctrl *n* = 3, Inv + E2V *n* = 4. **B**, 48 h estrogen treatment reduces the amount of newly generated neutrophils in the bone marrow (BM); Percentage of BrdU^+^PI^+^ cells out of total BM neutrophils (CD45^+^CD11b^+^Ly6G^+^) via flow cytometry analysis, Inv + Ctrl *n* = 3, Inv + E2V *n* = 4. **C**, 48 h estrogen treatment increases circulating LDN cell death; (i) Percentage of dead cells (Annexin V^+^ Dead stain^+^) out of total circulating LDN (CD45^+^CD11b^+^Ly6G^+^), (ii) Percentage of early apoptotic cells (Annexin V^+^ Dead stain^−^) out of total circulating LDN via flow cytometry analysis. Inv + Ctrl *n* = 3, Inv + E2V *n* = 4. **D-E**, Experiment is conducted according to the description in Fig. 1. **D**, Estrogen treatment led to a reduction in splenic neutrophils in tumour-bearing Null and Inv mice; Percentage of splenic neutrophils (CD45^+^CD11b^+^Ly6G^+^) out of viable CD45^+^ population via flow cytometry analysis, Null+Ctrl (N.T) *n* = 3, Null+Ctrl *n* = 5, Null+E2V *n* = 5, Inv + Ctrl *n* = 5, Inv + E2V *n* = 6. E, Estrogen treatment did not result in any significant changes in tumour neutrophils; Percentage of neutrophils (CD45^+^CD11b^+^Ly6G^+^) out of viable CD45^+^ population in tumour via flow cytometry analysis, Null+Ctrl *n* = 5, Null+E2V *n* = 5, Inv + Ctrl *n* = 5, Inv + E2V *n* = 6. Data represented as mean ± SEM. Statistical significances determined using two-tailed unpaired student’s T-test in (**A**-**C**); one-way ANOVA followed by post-hoc tukey test in (**D** and **E**)

We next determined the mechanism leading to the reduction of circulating LDN by E2V. OVX Inv mice bearing 4 T1-Luc2 tumour were treated with either Ctrl or E2V for 48 h and labelled with BrdU for 24 h prior to sample collection. The percentages of BrdU^+^ cells in the circulating LDN, HDN, and bone marrow neutrophils were evaluated using BrdU incorporation assay [17]. After 48 h E2V treatment, there were no significant differences in BrdU^+^ cells in the circulating LDN and HDN between Ctrl and E2V-treated groups (Fig. 4A). However, a significant decrease in BrdU^+^ neutrophils (CD45^+^CD11b^+^Ly6G^+^BrdU^+^PI^+^) in the bone marrow was observed (Fig. 4B). Newly synthesized neutrophils can remain in bone marrow for 4–6 days before moving to the circulation [18]. A reduction in mitotic neutrophils in the bone marrow suggests that there is a reduced LDN output.

LDN cell death was also determined by flow cytometry analysis of annexin V staining, which detect membrane phosphatidylserine of apoptotic cells on the outer surface [19]. Treatment with estrogen for 48 h resulted in a significant increase in Annexin V^+^ Dead stain^+^ cells (Fig. 4 Ci). However, we did not detect a significant increase in the percentage of early apoptotic cells (Annexin V^+^ Dead stain^−^) (Fig. 4Cii). It is possible that LDN apoptosis triggered after 48 h E2V treatment has already reached the late stages (Annexin V^+^ Dead stain^+^).

It has been reported that estrogen treatment increased both blood and splenic Ly6C^+^/Ly6G^+^ neutrophils in mice with lung or mammary tumour [12]. We looked into the possibility that estrogen-induced decrease of circulating LDN was resulted from the diversion of neutrophils into the spleen or tumour. We found ~ 50-folds increase in the percentage of splenic Ly6G^+^ neutrophils in tumour-bearing mice compared to tumour-free Null mice. This increase is greater than the increase in circulating neutrophils. With 14 days treatment of E2V, we observed a ~ 50% reduction of splenic neutrophils (CD45^+^CD11b^+^Ly6G^+^) in both tumour-bearing Null and Inv mice (Fig. 4D). This 50% reduction in neutrophils is higher than the percentage of reduction of LDN in the blood (~ 30 and 15% in Null and Inv mice, respectively).

There is a slight increase of tumour-associated neutrophils (CD45^+^CD11b^+^Ly6G^+^) in Null mice and decrease in Inv mice with E2V treatment, but the changes are not statistically significant (Fig. 4E). It is thus unlikely that the observed reduction in circulating LDN by E2V was due to increased mobilization into the tumour.

### Estrogen increases the expression of pro-tumoral markers in both circulating LDN and HDN of Inv mice

**Fig. 5:**
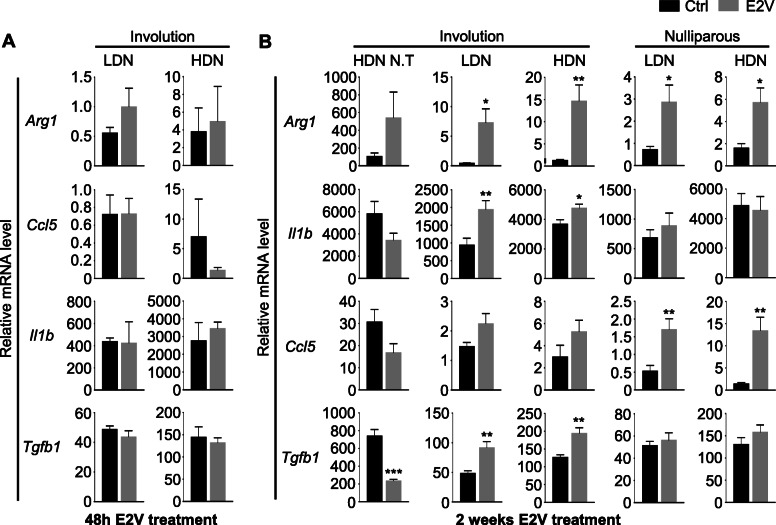
Extended estrogen treatment increases pro-tumoral markers expression in both circulating LDN and HDN of tumour-bearing mice. **a** Experiment is conducted according to the description in Fig. 3F-H. Treatment with estrogen for 48 h did not led to an increase in pro-tumoral markers in tumour-bearing Inv mice; Gene expression of pro-tumoral markers *Arg1*, *Il1b*, *Ccl5* and *Tgfb1* relative to *Gapdh* in isolated LDN and HDN by qPCR, Inv + Ctrl *n* = 3, Inv + E2V *n* = 3. **b** Experiment is conducted according to the description in Fig. 1. Gene expression of pro-tumoral markers *Arg1*, *Il1b*, *Ccl5,* and *Tgfb1* relative to *Gapdh* in isolated LDN and HDN by qPCR, Null+Ctrl *n* = 8, Null+E2V *n* = 8, Inv + Ctrl *n* = 11, Inv + E2V *n* = 12, Inv + Ctrl (N.T) *n* = 8, Inv + E2B (N.T) *n* = 4. Data represented as mean ± SEM. Statistical significances determined using two-tailed unpaired student’s T-test

Accumulation of LDN has been described in both the murine model and in breast cancer patients [7, 20, 21]. LDN are generally believed to be pro-tumoral, while its counterpart, the HDN was generally anti-tumoral. LDN’s pro-tumoral action mainly comes from its immune suppressive nature [8]. Since estrogen is known to elicit varying immunomodulating effect [22], we evaluated the effect of estrogen on the gene expression of known pro-tumoral genes, arginase-1 (*Arg1*), interleukin 1b (*Il1b*), and C-C motif chemokine ligand 5 (*Ccl5*) in neutrophils by qPCR. E2V treatment for 48 h had no significant effect on the expression of *Arg1*, *Il1b*, and *Ccl5* in both LDN and HDN (Fig. 5a). However, E2V treatment for 14 days resulted in increased expression of *Arg1*, *Il1b* in LDN and HDN of Inv mice, while *Ccl5*, *Arg1* were significantly upregulated in the LDN and HDN of Null mice (Fig. 5b). In non-tumour bearing Inv mice, only HDN were analysed because there were very few circulating LDN for isolation in non-tumour-bearing mice. E2V treatment for 14 days did not significantly affect the expression of these genes in neutrophils (Fig. 5b). Intriguingly, the relative expressions of all these genes are substantially higher in neutrophils of tumour-free mice than tumour-bearing mice.

Transforming growth factor beta (*Tgfb)* has been reported to have a pro-tumoral role in cancer development by promoting tumour metastasis and immune evasion [23]. Furthermore, it was also discovered that *Tgfb* can polarize tumour-associated neutrophil towards the pro-tumour phenotype [2, 24]. In this study, we observed that estrogen treatment for 14 days resulted in a significant increase in *Tgfb1* gene expression in both LDN and HDN of tumour-bearing Inv mice while having no effect in tumour-bearing Null mice. Interestingly, in non-tumour Inv mice, similar treatment instead led to a significant reduction of *Tgfb1* expression (Fig. 5b). Like the other genes tested, treatment for 48 h had no effect on *Tgfb1* expression in both LDN and HDN (Fig. 5a).

Taken together, one dose of E2V did not significantly influence the gene expression of circulating neutrophils, but estrogen exposure for 14 days enhanced the expression of putative pro-tumoral genes *Arg1*, *Il1b* and *Tgfb1* in Inv mice, potentially contributing to the increased tumour growth observed only in Inv mice (Fig. 1). In contrast, only *Ccl5* and *Arg1* is upregulated by E2V in Null mice.

## Discussion

With the understanding of neutrophils as important players in tumorigenesis in recent years, LDN emerges as a subclass of neutrophils of immune-suppressive and pro-tumoral properties [7, 9, 25]. It has been reported in both human and mice studies that the presence of tumour causes an increase in circulating neutrophils and in LDN [26, 27]. Consistent with the previous reports, the present study demonstrated a marked increase in the number of LDN in mice with tumours as compared to mice without tumours. Estrogen treatment during the course of tumour development (14 days) reduces the number of LDN levels by more than 10-folds in Inv and Null mice. The estrogen-induced reduction of LDN is associated with reduced number of BrdU^+^ neutrophils (CD45^+^CD11b^+^Ly6G^+^) in the bone marrow. This suggests that estrogen reduces LDN output from the bone marrow. This notion is supported by the observation that the effect of E2V on neutrophil reduction is accumulative over time. There is also a significant increase of apoptosis in LDN, which may be due to a shorter life span. These observations suggest that estrogen exerts inhibitory effect on neutrophil haematopoiesis under tumour-bearing condition.

Given that LDN is pro-tumoral [7, 9], estrogen-induced reduction of circulating LDN would be associated with an anti-tumoral effect. However, this is not the case. Whilst estrogen reduced LDN similarly in both Inv and Null mice, it promoted tumour growth in Inv mice but had no significant effect on Null mice. This difference in functional outcomes may be attributed to the difference in the tissue microenvironment. The pro-inflammatory microenvironment in the mammary gland of Inv mice facilitates estrogen-induced neutrophil recruitment and reprograming [15, 28], leading to the release of pro-tumoral cytokines/chemokines. This is in contrast with Null mice in which, estrogen is generally anti-inflammatory in the mammary tissue [29, 30]. Hence, the tissue microenvironment is an important determinant of neutrophil’s influence on tumour development. Additionally, there is also evidence of differences in estrogen regulation of gene expression of circulating neutrophils between Inv and Null mice. E2V upregulated the expression of pro-tumoral *Il1b* and *Tgfb1* in both LDN and HDN of Inv mice but had no effect in Null mice. The upregulation of *Tgfb1* is particularly interesting in this context because *Tgfb1* is known to induce the pro-tumoral properties of tumour-associated neutrophils. Inhibitor of TGFB1 receptor enhanced the infiltration and activity of anti-tumoral neutrophils in mesothelioma [2]. Similar observation was also reported in colon cancer [24]. The upregulation of *Tgfb1* in Inv mice by estrogen may provide more impetus for the pro-tumoral activity of neutrophils compared to the controls.

It is puzzling that the expression levels of these genes in control samples are ~ 5 to 10 folds higher in tumour-free mice than the tumour-bearing mice. Whilst the mechanism of decreased expression in tumour-bearing mice requires further investigation, the observation does indicate that tumour burden can reshape the epigenetics of neutrophils to alter gene expression. This would in turn change how neutrophils respond to tumour development. Neutrophils are known to undergo epigenetic changes during development and under mature state in response to environmental cues [31–33]. We have also reported that neutrophils in the post-weaning and nulliparous mammary tissue respond to estrogen differently [28]. Whether the innate immune function of neutrophils is generally compromised by tumour burden requires further investigation.

It is to be noted that estrogen has been reported to promote the survival and production of circulating neutrophils under non-tumour conditions. Treatment with physiological dose of estrogen for 24 h in human neutrophils was found to reduce neutrophil apoptosis [34]. In another spontaneous lupus-prone mice model, prolonged estrogen treatment for 7 to 8 weeks was also observed to increase circulating neutrophil number [35]. In addition, ERα antagonist inhibited in vitro differentiation of MDSCs by GM-CSF and IL6, suggesting stimulatory effect of estrogen [12]. This different effect of estrogen on mice under different conditions suggest that the tumour-host interaction can alter estrogen signalling in the bone marrow.

Taken together, this study reports that the prolonged estrogen exposure in tumour-bearing mice led to a marked reduction in circulating LDN. This effect is mediated, at least partly by inhibition of neutrophil output from the bone marrow and reduction of LDN lifespan. Estrogen also upregulated the expression of several known pro-tumoral markers in both HDN and LDN. Although the effect of estrogen on gene expressions appears to be correlated to its effect on tumour growth in Null and Inv mice, the mammary tissue microenvironment likely plays a significant role in shaping neutrophils activity. Whilst functional significance of estrogen-induced down-regulation of blood neutrophils is yet to be elucidated, the study highlights the potential of estrogenic compounds in modulating neutrophil biology in cancer patients. Tamoxifen, a widely used drug in ERα-positive breast cancer, is reported to function as a selective estrogen receptor agonist in neutrophils and hematopoietic progenitor cells [36, 37]. There have been reports of tamoxifen-induced neutropenia in breast cancer patients [38, 39], which may be caused by tamoxifen’s estrogenic activity in neutrophils. Estrogen levels in premenopausal cancer patients may also alter the levels and activities of LDN and HDN. Since neutrophils are the most abundant leucocytes, alteration of their activity may influence the outcome of the disease. Further investigation of how estrogen influences neutrophils activity in cancer patients may clarify the clinical relevance of the findings.

## Supplementary Information

**Additional file 1 **: **Supplementary Figure 1A**. Representative full flow cytometry analysis gating strategy. **Supplementary Figure 1B**. Representative gating strategy for Annexin V flow cytometry analysis. **Supplementary Figure 2**. Representative flow cytometry dot plot of circulating neutrophils out of viable CD45+ population. **Supplementary Figure 3**. Representative flow cytometry dot plot. A, Circulating monocytes (CD45+CD11b+Ly6G-hi) out of viable CD45+ population. B, Circulating CD4+ and CD8+ T-cells out of viable CD45+ population. **Supplementary Figure 4**. Representative flow cytometry dot plot of circulating LDN out of viable CD45+ population of the percoll isolated mononuclear cell fraction from blood.

## Data Availability

The datasets used and/or analysed during the current study are available from the corresponding author on reasonable request.

## Abbreviations

Ctrl: Control-treated
E2V: Estradiol valerate
HDN: High-density neutrophils
Inv: Post-weaning mammary involution
LDN: Low-density neutrophils
Null: Nulliparous
OVX: Ovariectomy
