# Supplementary material for: Estrogen markedly reduces circulating low-density neutrophils and enhances pro-tumoral gene expression in neutrophil of tumour-bearing mice
Source: BMC Cancer. 2021 Sep 11;21:1017. doi: 10.1186/s12885-021-08751-2 (PMC8436517; doi:10.1186/s12885-021-08751-2)
Supplement: Supplementary file 1 — Additional file 1 : Supplementary Figure 1A. Representative full flow cytometry analysis gating strategy. Supplementary Figure 1B. Representative gating strategy for Annexin V flow cytometry analysis. Supplementary Figure 2. Representative flow cytometry dot plot of circulating neutrophils out of viable CD45+ population. Supplementary Figure 3. Representative flow cytometry dot plot. A, Circulating monocytes (CD45+CD11b+Ly6G-hi) out of viable CD45+ population. B, Circulating CD4+ and CD8+ T-cells out of viable CD45+ population. Supplementary Figure 4. Representative flow cytometry dot plot of circulating LDN out of viable CD45+ population of the percoll isolated mononuclear cell fraction from blood. [file 12885_2021_8751_MOESM1_ESM.pptx]

## Slide 1
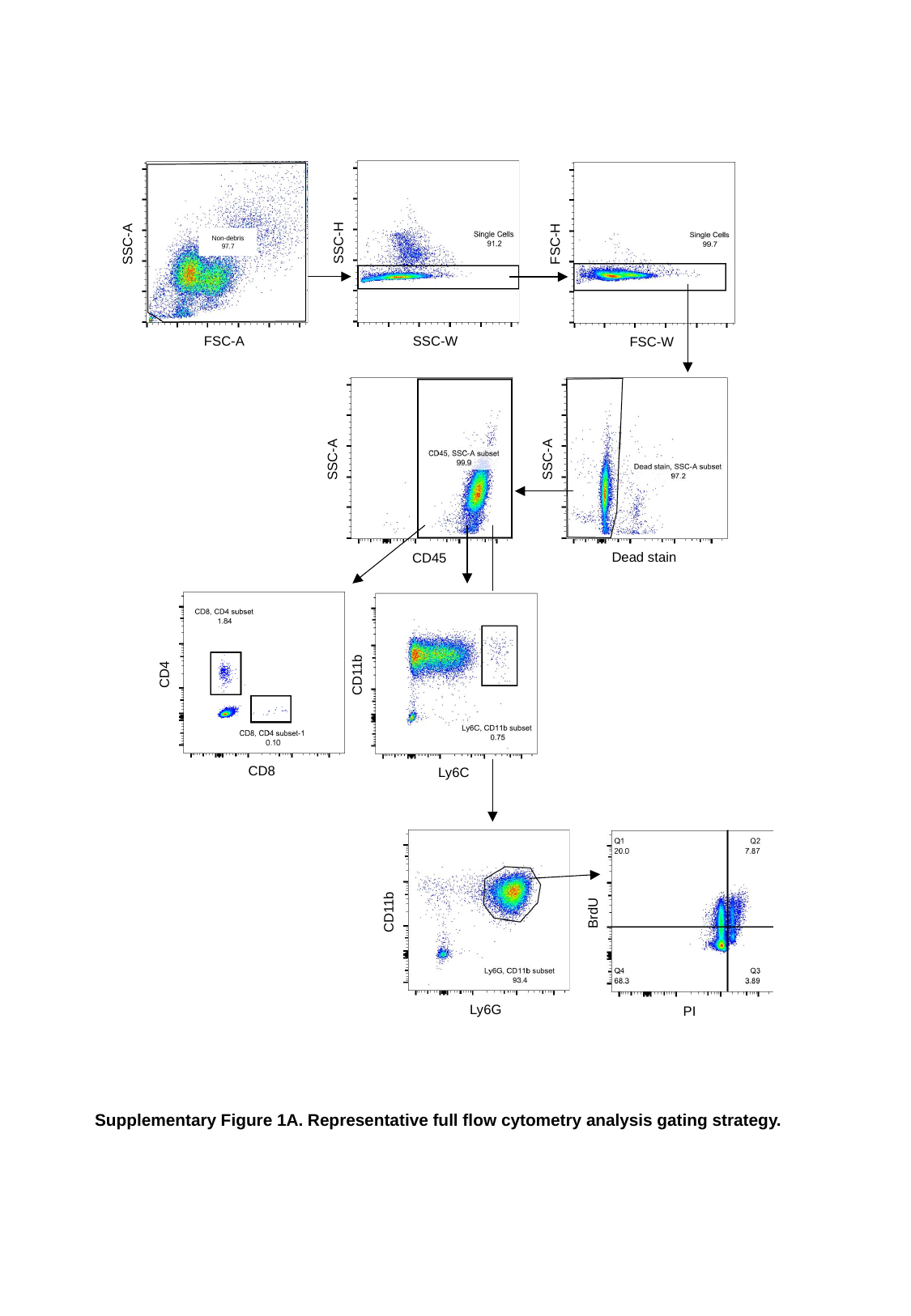

SSC-H
SSC-W
FSC-H
FSC-W
SSC-A
FSC-A
Non-debris
97.7
SSC-A
CD45
SSC-A
Dead stain
CD4
CD8
CD11b
Ly6C
CD11b
Ly6G
BrdU
PI
Supplementary Figure 1A. Representative full flow cytometry analysis gating strategy.

## Slide 2
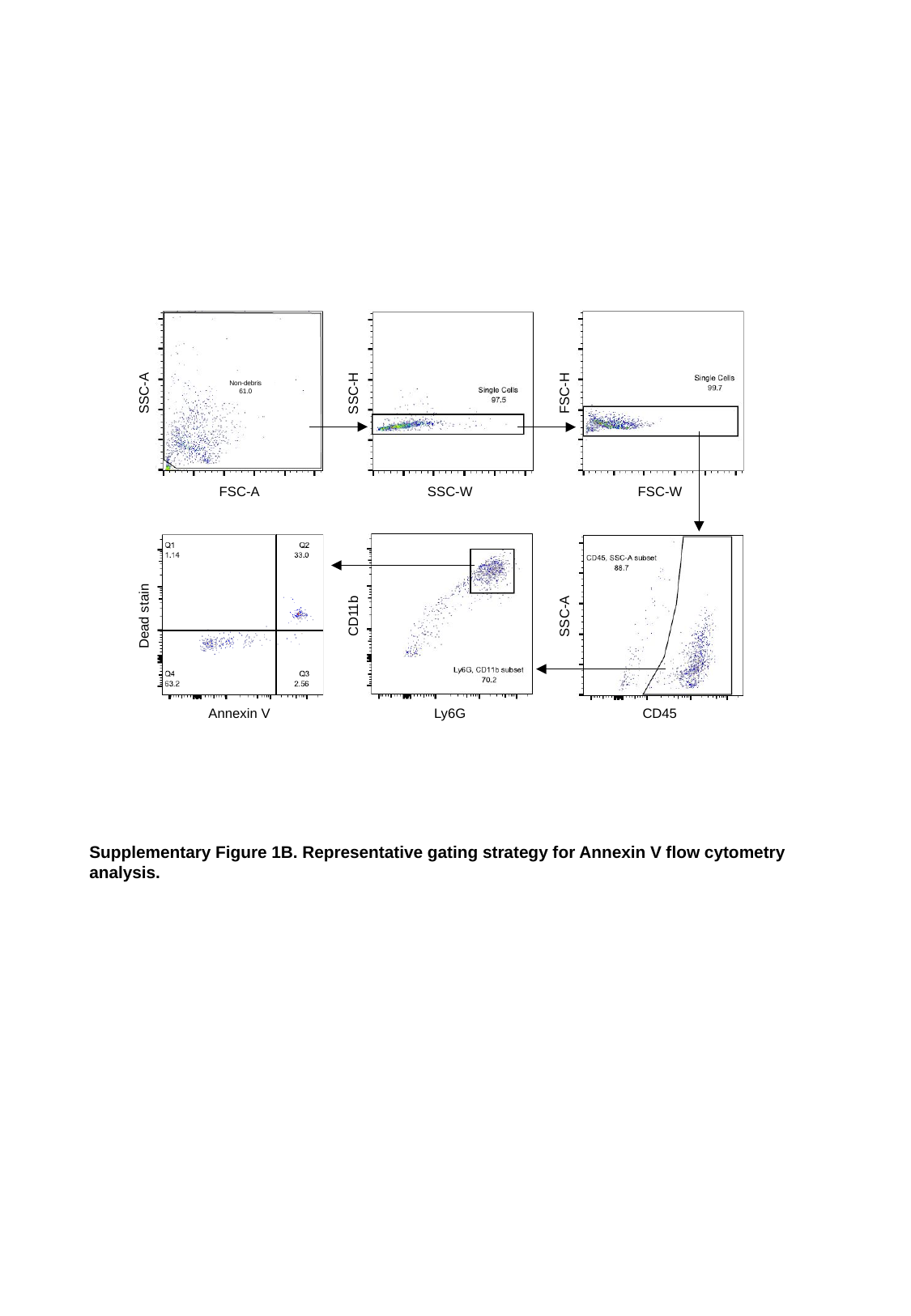

SSC-H
SSC-W
FSC-H
FSC-W
Non-debris
61.0
SSC-A
FSC-A
SSC-A
CD45
CD11b
Ly6G
Dead stain
Annexin V
Supplementary Figure 1B. Representative gating strategy for Annexin V flow cytometry analysis.

## Slide 3
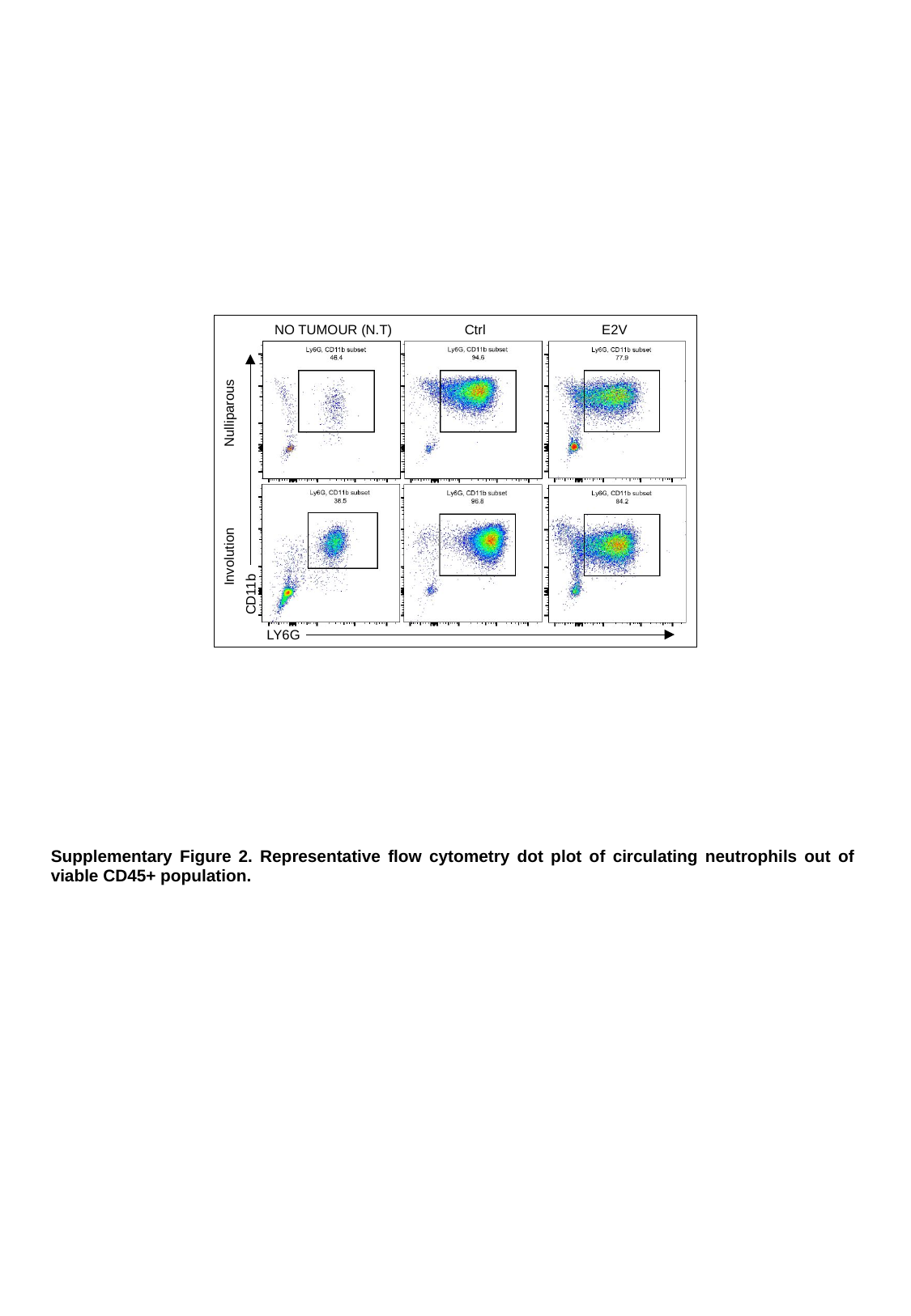

NO TUMOUR (N.T)
Ctrl
E2V
Nulliparous
Involution
CD11b
LY6G
Supplementary Figure 2. Representative flow cytometry dot plot of circulating neutrophils out of viable CD45+ population.

## Slide 4
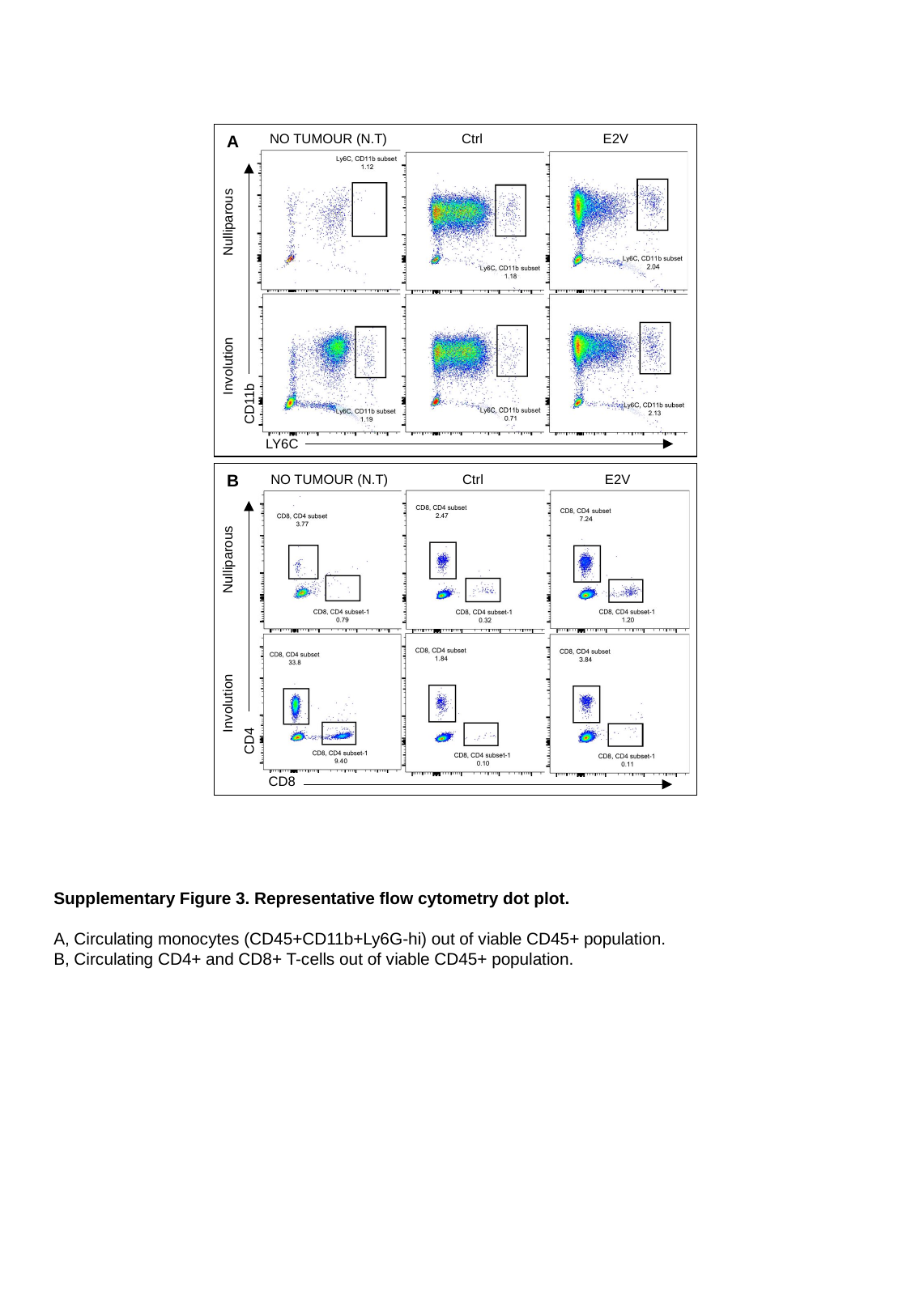

NO TUMOUR (N.T)
Ctrl
E2V
A
Nulliparous
Involution
CD11b
LY6C
B
NO TUMOUR (N.T)
Ctrl
E2V
Nulliparous
Involution
CD4
CD8
Supplementary Figure 3. Representative flow cytometry dot plot.
A, Circulating monocytes (CD45+CD11b+Ly6G-hi) out of viable CD45+ population.
B, Circulating CD4+ and CD8+ T-cells out of viable CD45+ population.

## Slide 5
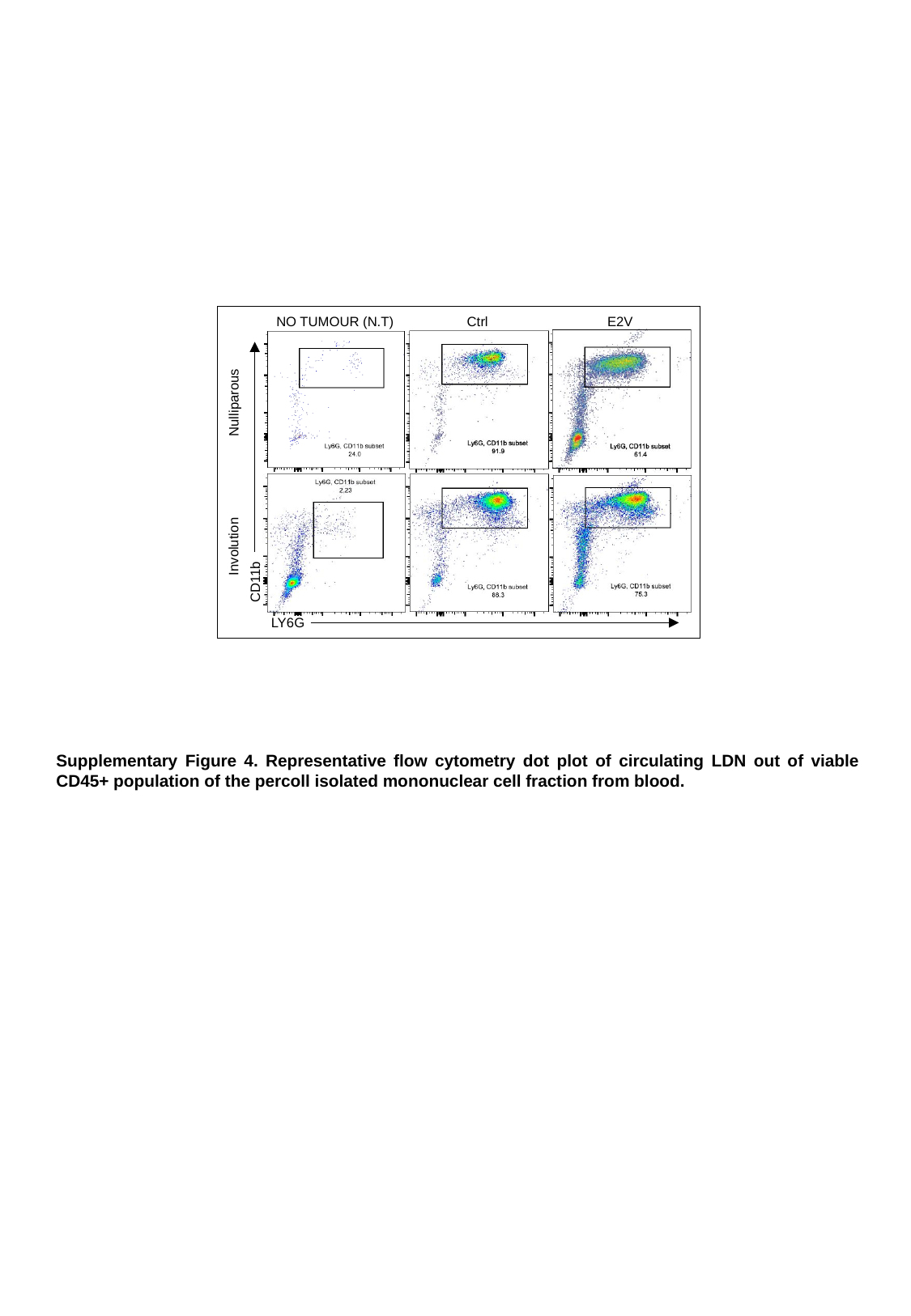

NO TUMOUR (N.T)
Ctrl
E2V
Nulliparous
Involution
CD11b
LY6G
Supplementary Figure 4. Representative flow cytometry dot plot of circulating LDN out of viable CD45+ population of the percoll isolated mononuclear cell fraction from blood.
